# LncRNA-AC02278.4 Is a Novel Prognostic Biomarker That Promotes Tumor Growth and Metastasis in Lung Adenocarcinoma

**DOI:** 10.3389/fonc.2022.860961

**Published:** 2022-04-06

**Authors:** Xi Chen, Fan Zhou, Wenjun Ren, Jishu Guo, Xiaobin Huang, Jun Pu, Xiaoqun Niu, Xiulin Jiang

**Affiliations:** ^1^ Department of Neurosurgery, The second Affiliated Hospital of Kunming Medical University, Kunming, China; ^2^ Hematology and Rheumatology Department, The Pu’er People’s Hospital, Pu’er, China; ^3^ Department of Cardiovascular Surgery, The First People’s Hospital of Yunnan Province, Kunming, China; ^4^ Institute for Ecological Research and Pollution Control of Plateau Lakes, School of Ecology and Environmental Sciences, Yunnan University, Kunming, China; ^5^ Department of Respiratory Medicine, Second Hospital of Kunming Medical University, Kunming, China; ^6^ Kunming College of Life Science, University of Chinese Academy of Sciences, Beijing, China

**Keywords:** lncRNA-AC02278.4, lung adenocarcinoma, prognosis, biomarker, cell proliferation, cell migration

## Abstract

LncRNA-AC02278.4 (ENSG00000248538) is a long non-coding RNA (lncRNA) found to be highly expressed in multiple human cancers including lung adenocarcinoma (LUAD). However, the underlying biological function and potential mechanisms of AC02278.4 driving the progression of LUAD remain unclear. In this study, we investigated the role of AC02278.4 in LUAD and found that AC02278.4 expression was significantly increased in datasets extracted from The Cancer Genome Atlas. Increased expression of lncRNA-AC02278.4 was correlated with advanced clinical parameters. Receiver operating characteristic (ROC) curve analysis revealed the significant diagnostic ability of AC02278.4 [area under the ROC curve (AUC) = 0.882]. In addition, gene set enrichment analysis (GSEA) enrichment showed that AC02278.4 expression was correlated with immune response-related signaling pathways. Finally, we determined that AC02278.4 regulated cell proliferation and migration of LUAD *in vitro*. Our clinical sample results also confirmed that AC02278.4 was highly expressed in LUAD and correlated with adverse clinical outcomes. In conclusion, our data demonstrated that AC02278.4 was correlated with progression and immune infiltration and could serve as a prognostic biomarker for LUAD.

## Introduction

Lung cancer is the leading cause of cancer-related death worldwide, according to cancer statistics 2020. The incidence rate of lung cancer ranks second, while the death rate of lung cancer ranks first (1). Lung cancer includes small cell lung carcinoma (SCLC) and non-SCLC (NSCLC). NSCLC includes lung adenocarcinoma (ADC) (LUAD), lung squamous cell carcinoma (SCC), and large cell lung carcinoma. NSCLC accounts for approximately 85% of all cases (2). Despite various treatments being applied during diagnostic and therapeutic procedures for lung cancer, the 5-year survival rate of lung cancer still remains poor (3). Therefore, elucidating the molecular mechanisms of lung oncogenesis and identifying new therapeutic targets or biomarkers are essential for effectively preventing the development of lung cancer.

The initiation and progression of lung cancer are very complicated processes that involve genetic mutations, tumor microenvironment, and the abnormal activation of epigenetic modification (4–6). Simultaneously, epigenetic changes in lung cancer such as histone modifications (7), DNA methylation (8), and non-coding RNAs (ncRNAs) (9) have been far and wide studied. Long ncRNAs (lncRNAs) are a class of RNA molecules longer than 200 nucleotides in length with considerable potential to be involved in cancer development (10, 11). Emerging evidence has confirmed that lncRNAs participated in modulating gene expression *via* cis or trans manner (12).

Various regulatory mechanisms for an lncRNA have been well established. LncRNAs can 1) affect downstream gene expression *via* inhibiting the activity of RNA polymerase II or affect chromatin remodeling and histone modification state; 2) modulate the mRNA splicing process *via* complementary binding with pre-mRNAs; 3) interact with protein and regulate protein activity; 4) function as scaffolds to promote RNA–protein complexes form; 5) modulate the subcellular localization of proteins; and 6) function as miRNA sponge to regulate gene expression (13). Moreover, accumulating evidence has confirmed that lncRNAs correlate with the pathogenesis of lung cancers through regulating the proliferation and invasion, cell cycle, cell autophagy, cell apoptosis, stemness of lung cancer stem cell, chemotherapy resistance, and tumor microenvironment (14). For example, Gong et al. found that lncRNA JPX was highly expressed in lung cancer and correlated with the tumor size and an advanced stage. Forced expression of JPX facilitated lung cancer cell proliferation *in vitro* and facilitated lung tumor growth *in vivo* (15). Lu et al. reported that lnc-IGFBP4-1 was overexpressed in lung cancer tissues and that its higher expression was associated with TNM stage and lymph node metastasis. Depletion of lnc-IGFBP4-1 significantly inhibited cell proliferation and induced apoptosis. Further research showed that lnc-IGFBP4-1 *via* affecting the expression of HK2, PDK1, and LDHA led to enhancing the ATP production level and is involved in lung cancer progression (16). Furthermore, lncRNA AFAP1-AS1 was found to modulated NSCLC cell proliferation *via* interacting with EZH2 and recruiting EZH2 to the promoter regions of p21, thus inhibiting p21 expression (17). However, the clinical value of lncRNA-AC02278.4 in LUAD has not been explored. Hence, this study aimed to investigate the role of lncRNA-AC02278.4 in the progression of LUAD.

In this study, we compared the expression of AC02278.4 between LUAD tissues and normal samples and explored the correlation between AC02278.4 expression and the clinical significance of LUAD. Furthermore, we explored the prognostic and diagnostic value of AC02278.4 in LUAD. Meanwhile, the correlation between AC02278.4 expression and immune infiltration was analyzed to explore the potential mechanisms involved in AC02278.4 modulation in the progression of LUAD. Finally, the biological role of AC02278.4 was identified in LUAD. In summary, we demonstrated the potential role of AC02278.4 in regulating tumor progression and its potential application in the diagnosis and prognostic evaluation in LUAD.

## Materials and Methods

### Data Collection

TCGA-LUAD cohort data and corresponding clinical information of 535 LUAD patients were downloaded from The Cancer Genome Atlas (TCGA) website (https://portal.gdc.cancer.gov/repository). LUAD patients were classified into low- and high-AC02278.4 expression groups according to the median AC02278.4 expression value. AC02278.4 expression data from GSE81089 datasets were downloaded from the Gene Expression Omnibus (GEO) database and validated for expression analyses. The gene expression profiles were normalized using the scale method provided in the “limma” R package. Data analysis was performed with the R (version 3.6.3) and ggplot2 [3.3.3] packages. The expression data were normalized to transcripts per kilobase million (TPM) values before further analysis. Besides, the receiver operating characteristic (ROC) curve was used to evaluate the diagnostic value of AC02278.4 using the pROC R package and ggplot2 R package.

### Nomogram Construction and Evaluation

Based on the multivariate Cox analysis results, a nomogram was established to predict the prognosis of LUAD patients. According to the prognosis model, each patient’s risk score was calculated as the total score of each parameter, which could predict the prognosis of LUAD patients (18).

### Gene Set Enrichment Analysis

The gene set enrichment analysis (GSEA) software was utilized to analyze the potential signaling pathway and molecular function in LUAD (19, 20). A customized Perl script was used to perform GSEA between high-AC02278.4 and low-AC02278.4 groups. According to the default statistical methods, an adjusted *p*-value <0.05 was considered significant.

### Immune Infiltration Analysis by Single-Sample Gene Set Enrichment Analysis

A GSVA R package was used to examine the LUAD immune infiltration of 24 tumor-infiltrating immune cells in tumor samples through single-sample gene set enrichment analysis (ssGSEA) (21, 22). The correlation between AC02278.4 and infiltration levels of immune cells was analyzed by Spearman’s correlation, and these immune cells with the different expression groups of AC02278.4 were analyzed by rank-sum test.

### Cell Culture

BEAS-2B cell line was purchased from Cell Bank of Kunming Institute of Zoology and cultured in BEGM media (Lonza, Basel, Switzerland; CC-3170). Lung cancer cell lines, including A549, H1299, and SPC-A1, were purchased from Cobioer (Nanjing, China) with short tandem repeat (STR) documents, and were cultured in RPMI-1640 medium (Corning, Manassas, VA, USA) supplemented with 10% fetal bovine serum (FBS) and 1% penicillin/streptomycin.

### Constructs, Lenti-Viral Preparation, and Establishment of Different Cell Lines

For shRNA knockdown experiments, independent shRNAs targeting a different region of AC02278.4 RNA were constructed using a pLKO.1 vector (Addgene, Cambridge, MA, USA), and the oligo sequences were provided as follows. Lentiviruses were generated according to the manufacturer’s protocol as previously documented (5), and indicated cells were infected by viruses twice with 48- and 72-h viral supernatants containing 4 μg/ml of polybrene, and stable cell lines were established by appropriate puromycin selection. The two independent AC02278.47 targeting sequences are as follows: shRNA#1, 5′-GGCACTTCGTGGCTGAACCGA-3′; shRNA#2, 5′-GGGGAACAATGGCTTCAGCAG-3′.

### Cell Proliferation Assays

For BrdU incorporation assay, indicated cells were cultured in 8-well chamber slides for 24 h, pretreated with or without SC79 (Beyotime, Shanghai, China; SF2730) for another 24 h and then treated with 10 µM of BrdU (Abcam, Cambridge, UK; ab142567) for 20 min. Subsequently, indicated cells were fixed with 4% paraformaldehyde (PFA) at room temperature for 20 min and then incubated with BrdU primary antibody (Abcam, ab6326) followed by secondary antibody detection. The cell nuclei were stained with DAPI as previously documented (5).

### Cell Migration Assays

Cell migration assay was performed as previously described (15). Briefly, indicated cells were seeded into 6-well plates (9 × 10^5^/cell) and incubated for 1 day, and then a straight line was scraped with pipette tips. Detached cells were removed. Photographs were taken at the indicated time, and the relative traveled distance was measured. For the trans-well migration assay, 2.5 × 10^4^ cells/well in 100 μl of serum-free medium were plated in a 24-well plate chamber insert, and the lower chamber was filled with 10% FBS. After incubation for 24 h, cells were fixed with 4% PFA, washed, and then stained with 0.5% crystal violet for further imaging.

### Real-time RT-PCR assay

In the real-time RT-PCR assay, cells were lysed by RNAiso Plus (Takara Bio, Beijing, China; Cat. 108-95-2). Total RNAs were extracted according to the manufacturer’s protocol and then reverse transcribed by using RT reagent Kit (Takara Bio, Beijing, China). The primer used in this study is as follows: β-actin-F: AAGTGTGACGTGGACATCCGC, β-actin-R: CCGGACTCGTCATACTCCTGCT, AC02278.4-F: TCAAGGCATCATGTGTCATT, AC02278.4-R: ACCTGCTGAAGCCATTGTTCC.

### Statistical Analysis

For the datasets from TCGA database, statistical analyses were performed using R. The Wilcoxon rank-sum test and chi-square test were used to estimate the association between AC02278.4 and clinical pathologic characteristics. The Kaplan–Meier method was used to calculated LUAD patient survival rates. Univariate and multivariate Cox analyses were performed to assess the correlation between clinical features and overall survival (OS), disease-free survival, and progression-free survival (PFS). For the data regarding the function of AC02278.4, Graph Pad Prism 7.0 was used for statistical analyses. Student’s *t*-test evaluated the statistical significance between groups. The significance of the data between the two experimental groups was determined by Student’s *t*-test, and multiple group comparisons were analyzed by one-way ANOVA. *p* < 0.05 (*), *p* < 0.01 (**), and *p* < 0.001 (***), were significant.

## Results

### AC02278.4 Was Overexpressed in Lung Adenocarcinoma

**Figure 1:**
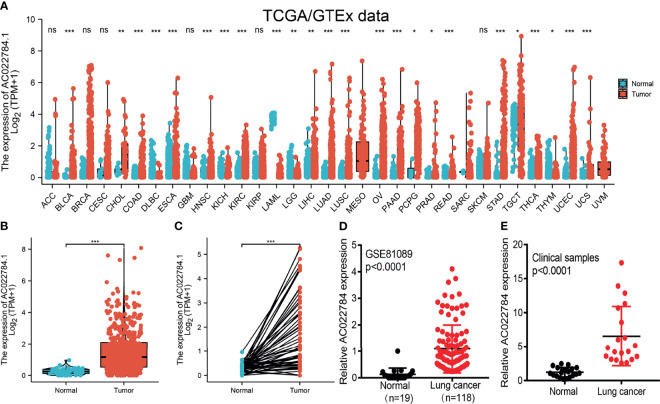
LncRNA-AC02278.4 was highly expressed in lung adenocarcinoma. **(A)** Expression of lncRNA-AC02278.4 in diverse human cancer based on the TCGA and GTEx datasets. **(B)** Expression of lncRNA-AC02278.4 in lung cancer based on TCGA dataset. **(C)** Expression levels of lncRNA-AC02278.4 in 59 paired adjacent normal tissues and paired samples. **(D)** Expression levels of lncRNA-AC02278.4 in lung cancer based on the GEO dataset. **(E)** Relative lncRNA-AC02278.4 expression detected by RT-qPCR in 20 paired lung cancer and non-cancerous tissues. ACC, adrenocortical carcinoma; BLCA, bladder urothelial carcinoma; BRCA, breast invasive carcinoma; CESC, cervical squamous cell carcinoma and endocervical adenocarcinoma; CHOL, cholangiocarcinoma; COAD, colon adenocarcinoma; DLBC, lymphoid neoplasm diffuse large B-cell lymphoma; ESCA, esophageal carcinoma; GBM, glioblastoma multiforme; HNSC, head and neck squamous cell carcinoma; KICH, kidney chromophobe; KIRC, kidney renal clear cell carcinoma; KIRP, kidney renal papillary cell carcinoma; LAML, acute myeloid leukemia; LGG, brain lower-grade glioma; LIHC, liver hepatocellular carcinoma; LUAD, lung adenocarcinoma; LUSC, lung squamous cell carcinoma; MESO, mesothelioma; OV, ovarian serous cystadenocarcinoma; PAAD, pancreatic adenocarcinoma; PCPG, pheochromocytoma and paraganglioma; PRAD, prostate adenocarcinoma; READ, rectum adenocarcinoma; SARC, sarcoma; SKCM, skin cutaneous melanoma; STAD, stomach adenocarcinoma; TGCT, testicular germ cell tumors; THCA, thyroid carcinoma; THYM, thymoma; UCEC, uterine corpus endometrial carcinoma; UCS, uterine carcinosarcoma; UVM, uveal melanoma; TCGA, The Cancer Genome Atlas; GTEx, Genotype-Tissue Expression; GEO, Gene Expression Omnibus. NS, *p* > 0.05,*p < 0.05, **p < 0.01, ***p < 0.001.

We simultaneously analyzed the expression profiles of AC02278.4 (ENSG00000248538) based on TCGA database. The results confirmed that AC02278.4 was highly expressed in multiple cancer types compared to the normal samples (**Figure 1A**). To examine the expression of AC02278.4 in LUAD and normal samples, we analyzed the expression of AC02278.4 in 535 tumor tissues and 59 normal prostate tissues of TCGA data, and we uncovered that AC02278.4 was upregulated in LUAD tissues (**Figure 1B**). There were 59 pairs of LUAD cancer samples and matched adjacent normal samples in TCGA data. The expression level of AC02278.4 was also higher in LUAD samples than matched adjacent normal samples (**Figure 1C**). Moreover, we found that AC02278.4 increased in lung cancer tissues by analyzing the GEO dataset (**Figure 1D**). To validate the expression of AC02278.4 in LUAD, we first employed qRT-PCR assay to detect the expression of AC02278.4 in 20 pairs of LUAD tissues and matched adjacent normal tissues. It was identified that AC02278.4 was significantly upregulated in LUAD tissue than adjacent normal tissue (**Figure 1E**).

### Overexpression of AC02278.4 Was Associated With Adverse Clinical Parameters in Lung Adenocarcinoma

**Figure 2:**
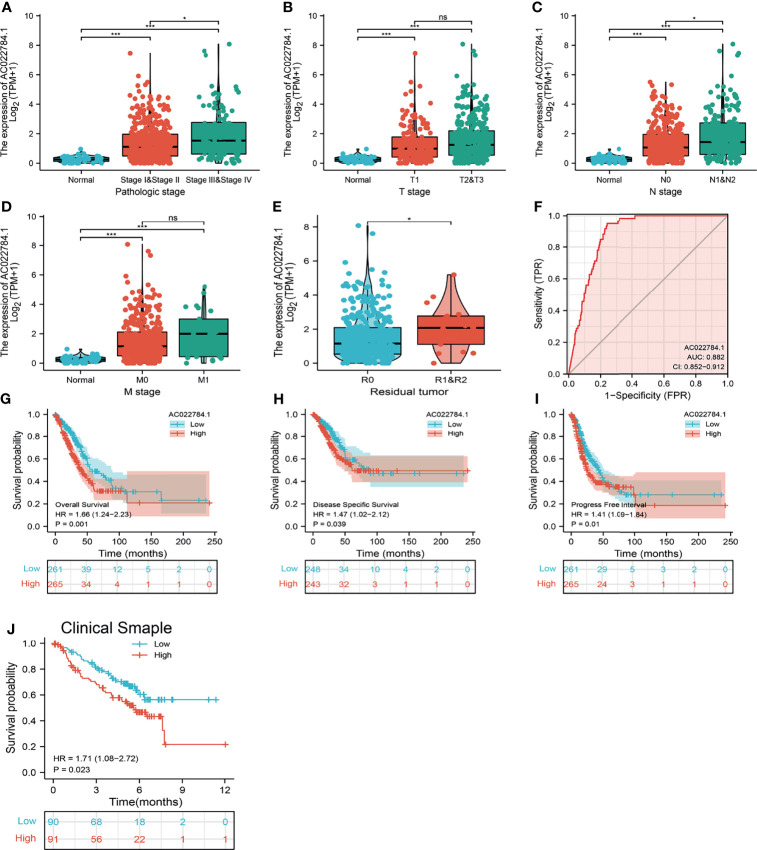
Clinical significance of lncRNA-AC02278.4 in lung adenocarcinoma. Correlation between lncRNA-AC02278.4 expression and clinical parameters, including **(A)** pathological stage, **(B–D)** TNM stage, and **(E)** residual tumor. **(F)** ROC curves were used to determine the diagnostic value of lncRNA-AC02278.4 in lung adenocarcinoma. **(G–J)** Kaplan–Meier survival curves showed that lung adenocarcinoma patients with high lncRNA-AC02278.4 expression exhibited poor overall survival, disease-specific survival, and progression-free survival of AC02278.4 in LUAD as determined by TCGA-LUAD dataset and clinical samples. NS, *p* > 0.05, *p < 0.05, ***p < 0.001. ROC, receiver operating characteristic; LUAD, lung adenocarcinoma.

To examine the clinical relevance of AC02278.4 in LUAD, 535 LUAD patients with clinical parameters were classified into two subgroups based on the mean value of relative AC02278.4 expression. We then explored the correlations between AC02278.4 expression and clinical parameters, including pathologic stage, TNM stage, and residual tumor. Regarding the tumor pathological stage, a significant increase in AC02278.4 expression was observed in LUAD patients in stages 1, 2, 3, and 4 (**Figure 2A**). Based on the cancer stage, AC02278.4 expression was higher in patients with LUAD classified as T stage (T1, T2, and T3), N stage (N0, N1, and N2), and M stage (M0 and M1) (**Figures 2B**–**D**). We also found that AC02278.4 expression was significantly correlated with the residual tumor stage (**Figure 2E**). Furthermore, ROC analysis showed that the AC02278.4 could be used to differentiate LUAD patients from normal control with a specificity (AUC = 0.882) (**Figure 2F**). Additionally, Kaplan–Meier analysis showed that LUAD patients with higher AC02278.4 expression were associated with poor OS, disease-free survival, and PFS (**Figures 2G**–**I**). To further validate the correlation between AC02278.4 expression and OS, we examined the prognosis of AC02278.4 in lung cancer by clinical samples from Yunnan Cancer Hospital (N = 181). The results also showed that high AC02278.4 expression had a worse OS than low AC02278.4 expression (**Figure 2J**).

### Univariate and Multivariate Cox Regression Analyses of Different Parameters on Overall Survival

We conducted a univariate Cox regression analysis in the TCGA-LAUD cohort to determine whether AC02278.4 expression level and pathologic stage might be valuable prognostic biomarkers. In the univariate Cox regression analysis, high expression of AC02278.4, pathologic stage, and TNM stage were associated with OS in LUAD patients. To ascertain whether AC02278.4 expression level could be an independent prognostic factor for patients with LUAD, a multivariate Cox regression analysis was performed. We confirmed that increased AC02278.4 expression was a significant independent prognostic factor in the TCGA-LAUD cohort that directly correlated with adverse clinical outcomes, along with T stage (**Table 1**).

**Table 1:** Univariate and multivariate Cox regression analyses of different parameters on overall survival in lung adenocarcinoma.

Characteristics	Total (N)	Univariate analysis		Multivariate analysis	
		Hazard ratio (95% CI)	*p*-Value	Hazard ratio (95% CI)	*p*-Value
T stage	523				
T1 and T2	457				
T3 and T4	66	2.317 (1.591–3.375)	<0.001	1.638 (1.018–2.635)	0.042
N stage	510				
N0 and N1	437				
N3 and N2	73	2.321 (1.631–3.303)	<0.001	1.293 (0.626–2.674)	0.488
Pathologic stage	518				
Stage II and stage I	411				
Stage IV and stage III	107	2.664 (1.960–3.621)	<0.001	1.802 (0.839–3.871)	0.131
M stage	377				
M0	352				
M1	25	2.136 (1.248–3.653)	0.006	1.192 (0.541–2.626)	0.664
AC022784 1	526	1.251 (1.141–1.372)	<0.001	1.168 (1.053–1.296)	0.003

### Construction and Validation of AC02278.4-Based Nomogram

**Figure 3:**
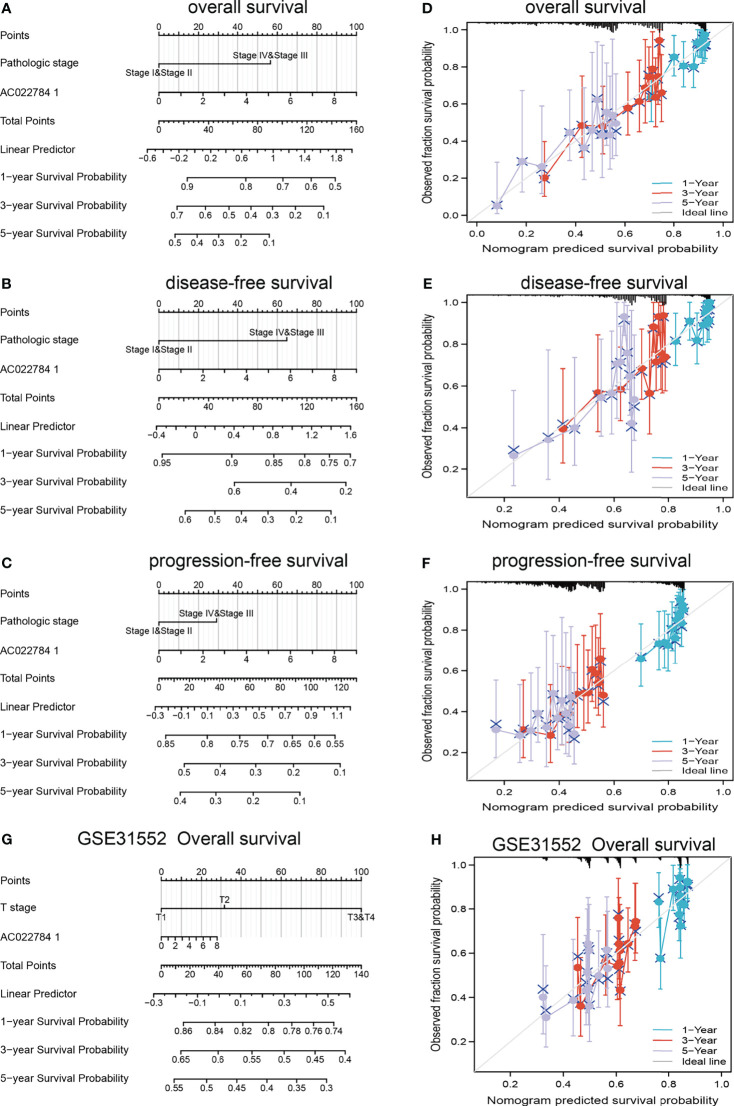
Construction and performance validation of the lncRNA-AC02278.4-based nomogram for lung adenocarcinoma patients. Nomogram to predict the **(A)** overall survival, **(B)** disease-specific survival, and **(C)** progression-free survival for lung cancer patients. The calibration curve and Hosmer–Lemeshow test of nomograms in the TCGA-lung adenocarcinoma cohort for **(D)** overall survival, **(E)** disease-specific survival, and **(F)** progression-free survival. **(G, H)** Using GEO dataset to validation of the lncRNA-AC02278.4-based nomogram for lung adenocarcinoma patients. GEO, Gene Expression Omnibus.

The multivariate analysis result confirmed that AC02278.4 is an independent prognostic factor in LUAD. We then constructed a prediction model for OS, disease-free survival, and PFS by integrating AC02278.4 expression and pathologic stage. We established a nomogram to integrate AC02278.4 as a LUAD biomarker; higher total points on the nomogram for OS, progression-free interval (PFI), and disease-specific survival (DSS) indicated a worse prognosis (**Figures 3A–E**). A higher point on the nomogram represented a worse prognostic factor.

Based on the calibration curve of nomograms for OS, DSS, and PFS, the predictions conformed well to observations in all patients, and the test showed no deviation from the perfect fit. The nomogram had a C-index of 0.657 and contained 1,000 bootstrap replicates [95% CI: (0.634–0.679)] for OS. We also found DSS [C-index: 0.594, CI: (0.573–0.615)] and PFS (C-index: 0.645, CI: 0.617–0.673). It was found that the bias-corrected line in the calibration plot was close to the ideal curve, indicating a strong correlation between predicted values and observed values (**Figures 3D–F**). In summary, these results indicated that the nomogram can well predict short- or long-term survival of LUAD patients. We also used the GEO dataset to validate the above result, as is shown in **Figures 3G, H**; using the GSE31552 dataset, we found that this nomogram could well predict the OS of LUAD patients.

### AC02278.4-Related Signaling Pathways Based on Gene Set Enrichment Analysis

**Figure 4:**
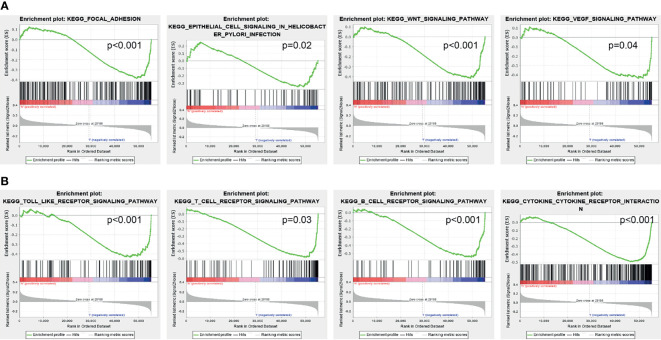
Identification of lncRNA-AC02278.4-related signaling pathways in lung adenocarcinoma. **(A, B)** Identification of lncRNA-AC02278.4-related signaling pathways by GSEA software. GSEA, gene set enrichment analysis.

GSEA was used to identify potential signaling pathways that are activated by high AC02278.4 expression. As shown in **Figures 4A, B**, there were eight significant signaling pathways related to the high AC02278.4 expression phenotype, including the focal adhesion, epithelial cell signaling in *Helicobacter pylori* infection, Wnt signaling pathway, VEGF signaling pathway, toll-like receptor signaling pathway, T-cell receptor signaling pathway, B-cell receptor signaling pathway, and cytokine–cytokine receptor interaction (**Figures 4A, B**).

### Correlation Between AC02278.4 Expression and Immune Infiltration

**Figure 5:**
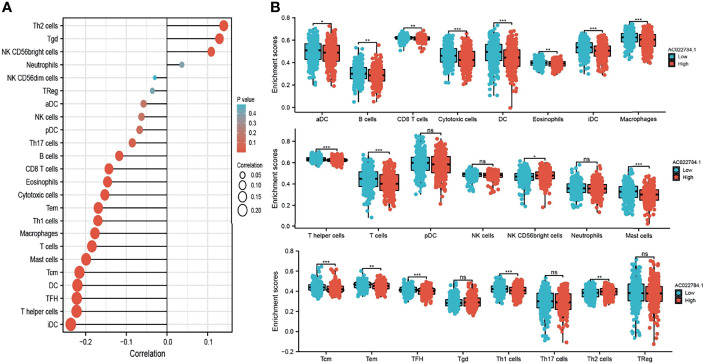
Correlation analysis of lncRNA-AC02278.4 expression and infiltration levels of immune cells in LUAD tissues. **(A)** Correlation between the relative abundances of 24 immune cells and lncRNA-AC02278.4 expression level. **(B)** Box plots of the correlations between lncRNA-AC02278.4 or molecular model expression and infiltration levels of immune cells. NS, *p* > 0.05,*p < 0.05, **p < 0.01, ***p < 0.001. LUAD, lung adenocarcinoma.

Using the ssGSEA method, we explored the correlation between AC02278.4 expression and infiltrating immune cells in LUAD. These results confirmed that AC02278.4 expression was negatively associated with infiltration levels of Th17 cells, B cells, CD8 T cells, eosinophils, cytotoxic cells, Tem, Th1 cells, macrophages, T cells, mast cells, Tcm, DC, TFH, T helper cells, and iDC (*p* < 0.001) and was positively correlated with that of Th2 cells, Tgd, and NK CD56bright cells (**Figure 5A**). Furthermore, we found that patients with AC02278.4 high-expression group showed a reduction in the numbers of infiltrating aDC, B cells, CD8 T cells, cytotoxic cells, DC, eosinophils, iDC, macrophages, T helper cells, T cells, mast cells, Tcm, Tem, TFH, and Th1 cells (**Figure 5B**).

### AC02278.4 Promotes Proliferation, Migration, and Invasion of Lung Adenocarcinoma Cells *In Vitro*

**Figure 6:**
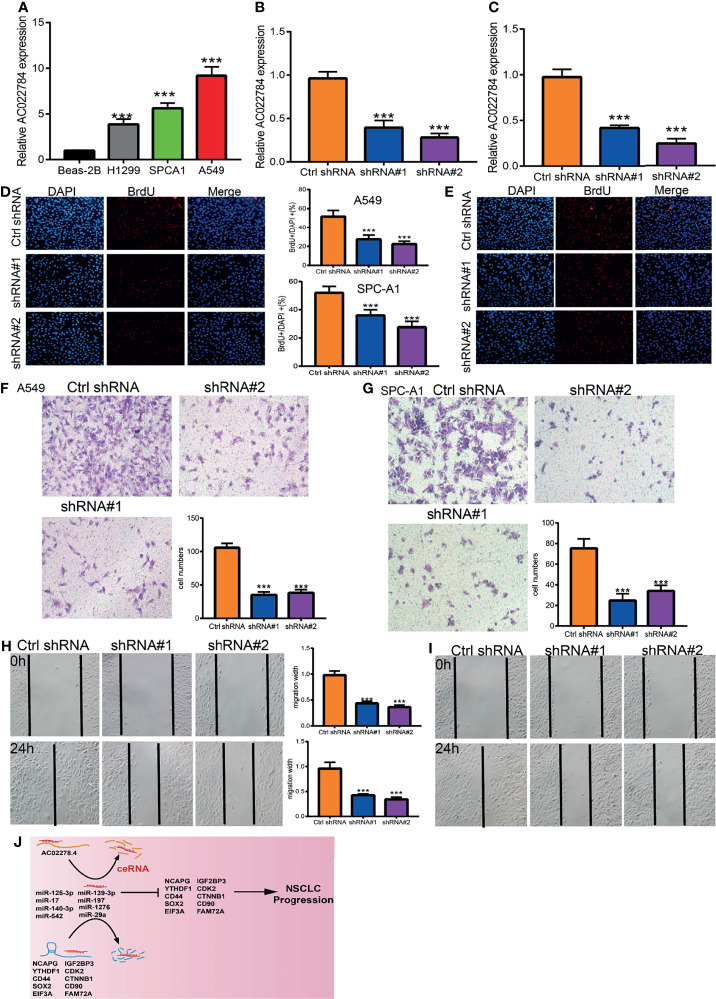
LncRNA-AC02278.4 promotes LUAD cell proliferation, migration, and invasion *in vitro*. **(A)** The relative expression level of lncRNA-AC02278.4 in lung adenocarcinoma cancerous cell lines, including H1299, SPCA1, and A549 examined by real-time RT-PCR, compared to normal human bronchial epithelial cell line: BEAS-2B. **(B, C)** Establishment of lncRNA-AC02278.4 knockdown cell lines in A549 and H1299 verified by real-time RT-PCR. **(D, E)** Knockdown of lncRNA-AC02278.4 significantly inhibits cell proliferation in A549 and H1299 cells, as measured with a BrdU Alexa Fluor Imaging Kit. Scale bar: 50 μm. **(F–I)** Knockdown of lncRNA-AC02278.4 dramatically inhibits A549 and H1299 cell migration’s ability as examined by transwell and wound healing assays. **(J)** The network of lncRNA–miRNA–mRNA. ***p < 0.001. LUAD, lung adenocarcinoma.

The above studies indicated that AC02278.4 expression was distinctly upregulated in LUAD tissues, and AC02278.4 might influence the progression in LUAD. To further investigate the biological role of AC02278.4 in LUAD, we first confirmed that the expression of AC02278.4 was significantly upregulated in H1299, A549, and SPC-A1 lung cancer cell lines compared to the human bronchial epithelial cells (BEAS2B) (**Figure 6A**). Furthermore, specific siRNA for AC02278.4 was used to construct A549 and SPC-A1 cells with stable knockdown of AC02278.4 expression. The knockdown efficiencies in transformed cell lines were detected by qRT-PCR analysis (**Figures 6B, C**). It was confirmed that knockdown of AC02278.4 reduced the proliferative capacity of A549 and SPC-A1 cells (**Figures 6D, E**) upon BrdU assays. Moreover, transwell assay and wound healing revealed that the migration and invasion abilities of A549 and SPC-A1 cells were significantly inhibited through downregulating AC02278.4 expression level (**Figures 6F**–**I**). Using various bioinformatics tools, we also constructed an mRNA–miRNA–lncRNA network; further study should be conducted to verify this result (**Figure 6J**). Briefly, it was suggested that AC02278.4 plays the role of an oncogene in lung cancer cells.

## Discussion

Recent studies consistently report that lncRNAs can modulate various hallmarks of lung cancer, including cancer cell proliferation, metabolic reprogramming, angiogenesis, cancer cell invasion and metastasis, and immunosurveillance (23, 24). It has been shown that lncRNAs may be effective and specific molecular markers for lung cancer diagnosis. For example, Tan et al. found that Linc00152 has an AUC value of 0.742 and may serve as a diagnostic marker distinguishing NSCLC (25). Furthermore, univariate and multivariate analyses as standard and reliable statistical methods are utilized to confirm whether an lncRNA can be regarded as an independent tumor marker for predicting the prognosis of lung cancer patients. By univariate and multivariate analyses, some lncRNAs have also been identified as independent prognostic markers in lung cancer. For instance, EGFR−AS1 was shown to be increased in NSCLC and correlated with a poor prognosis. With forced EGFR−AS1 expression promoting NSCLC cell proliferation and chemoresistance *via* regulating miR-223/IGF1R axis, univariate and multivariate analyses reported EGFR−AS1 as an independent prognostic marker in lung cancer (26).

Combining bioinformatics analyses, we found that AC02278.4 was highly expressed in LUAD, which was also correlated with OS, disease-free survival, and PFS in the LUAD patients of TCGA data. Our clinical sample analysis demonstrated that AC02278.4 was overexpressed in lung cancer. We also verified that increased AC02278.4 expression was a significant independent prognostic factor in the TCGA-LUAD cohort that directly correlated with adverse clinical outcomes. We also established a nomogram to integrate AC02278.4 as a LUAD biomarker; higher total points on the nomogram for OS, PFI, and DSS indicated a worse prognosis.

In this study, we investigated the underlying mechanisms through which AC02278.4 affected the progression of LUAD. GSEA enrichment confirmed that AC02278.4 was significantly associated with focal adhesion, epithelial cell signaling in *H. pylori* infection, Wnt signaling pathway, VEGF signaling pathway, toll-like receptor signaling pathway, T-cell receptor signaling pathway, B-cell receptor signaling pathway, and cytokine–cytokine receptor interaction, which indicated that AC02278.4 might have a crucial role in immune-response regulation and cell proliferation. Furthermore, we also explored the correlation between AC02278.4 expression and diverse immune infiltration levels in LUAD.

In this finding, we found that AC02278.4 expression was negatively associated with infiltration levels of Th17 cells, B cells, CD8 T cells, eosinophils, cytotoxic cells, Tem, Th1 cells, macrophages, T cells, mast cells, Tcm, DC, TFH, T helper cells, and iDC (*p* < 0.001) and was positively correlated with those of Th2 cells, Tgd, and NK CD56bright cells and thus plays an oncogenic role in LUAD. *In vitro*, depletion of AC02278.4 in A549 and SPC-A1 cells inhibited cell proliferation and migration. Based on the above findings, we proposed that AC02278.4 exerts an essential function in regulating the pathologic progression of LUAD.

This study improves our understanding of the correlation between lncRNA-AC02278.4 and LUAD, but some limitations still exist. First, although we explored the correlation between AC02278.4 and immune infiltration in LUAD patients, there is a lack of experiments to validate the function of AC02278.4 in the tumor microenvironment regulation of LUAD. Second, we uncover that depletion of AC02278.4 inhibits cell proliferation and cell migration of LUAD cells. However, the potential molecular mechanisms of AC02278.4 in cancer progression need to be explored in further studies. Third, we did not conduct the *in vivo* experiments to validate the function of AC02278.4 in the tumor metastasis and tumor microenvironment regulation of LUAD. In the future, we will pay more attention to the function of AC02278.4 in tumor metastasis and tumor microenvironment regulation of LUAD. Furthermore, we will perform more *in vivo* and *in vitro* experiments to explore the function and the potential molecular mechanisms of AC02278.4 in tumor metastasis and tumor microenvironment regulation of LUAD.

Overall, our results confirmed that AC02278.4 could serve as a potential novel prognostic biomarker for LUAD. Moreover, we explored the underlying evidence indicating that AC02278.4 regulates immune cell infiltration in the tumor microenvironment in LUAD patients. Therefore, these findings are potentially valuable in advancing our current understanding of not only the role of AC02278.4 but also its translational use in LUAD prognosis and immunotherapy.

## Conclusion

In summary, our findings provide the first evidence that AC02278.4 has significantly increased expression in LUAD and may serve as a promising diagnostic and prognostic biomarker for LUAD.

## Data Availability Statement

The original contributions presented in the study are included in the article/supplementary material. Further inquiries can be directed to the corresponding authors.

## Author Contributions

XC, FZ, WR and JG designed this work and performed related assay. XH analyzed the data. JP, XN and XJ supervised and wrote the manuscript. All authors have read and approved the final version of the manuscript.

## Funding

This work was supported by the Applied Basic Research Project of Yunnan Provincial Science and Technology Department and Kunming Medical University, No.2020001AY070001-117. The Open Project of The First People's Hospital of Yunnan Province Clinical Medicine Center (2021LCZXXF‐XZ03).

## Conflict of Interest

The authors declare that the research was conducted in the absence of any commercial or financial relationships that could be construed as a potential conflict of interest.

## Publisher’s Note

All claims expressed in this article are solely those of the authors and do not necessarily represent those of their affiliated organizations, or those of the publisher, the editors and the reviewers. Any product that may be evaluated in this article, or claim that may be made by its manufacturer, is not guaranteed or endorsed by the publisher.
