# Corrigendum: LncRNA-AC02278.4 is a novel prognostic biomarker that promotes tumor growth and metastasis in lung adenocarcinoma

**DOI:** 10.3389/fonc.2024.1390669

**Published:** 2024-03-13

**Authors:** Xi Chen, Fan Zhou, Wenjun Ren, Jishu Guo, Xiaobin Huang, Jun Pu, Xiaoqun Niu, Xiulin Jiang

**Affiliations:** ^1^ Department of Neurosurgery, The second Affiliated Hospital of Kunming Medical University, Kunming, China; ^2^ Hematology and Rheumatology Department, The Pu’er People’s Hospital, Pu’er, China; ^3^ Department of Cardiovascular Surgery, The First People’s Hospital of Yunnan Province, Kunming, China; ^4^ Institute for Ecological Research and Pollution Control of Plateau Lakes, School of Ecology and Environmental Sciences, Yunnan University, Kunming, China; ^5^ Department of Respiratory Medicine, Second Hospital of Kunming Medical University, Kunming, China; ^6^ Kunming College of Life Science, University of Chinese Academy of Sciences, Beijing, China

**Keywords:** lncRNA-AC02278.4, lung adenocarcinoma, prognosis, biomarker, cell proliferation, cell migration

In the published article, an author name was incorrectly written as Xin Chen. The correct spelling is Xi Chen.

The authors apologize for this error and state that this does not change the scientific conclusions of the article in any way. The original article has been updated.

